# Evaluation of the clinical value of heart rate variability in predicting vasovagal syncope

**DOI:** 10.3389/fcvm.2025.1684990

**Published:** 2026-01-21

**Authors:** Yueerguli Yusufuaji, Baopeng Tang, Li Men, Long Yang, Zulifeiya Musha, Ping Fan

**Affiliations:** 1 Department of Heart Function, First Affiliated Hospital of Xinjiang Medical University, Urumqi, Xinjiang, China; 2 Department of Cardiac Pacing and Electrophysiology, First Affiliated Hospital of Xinjiang Medical University, Urumqi, Xinjiang, China; 3 Xinjiang Key Laboratory of Cardiac Electrophysiology and Remodeling, First Affiliated Hospital of Xinjiang Medical University, Xinjiang, Urumqi, China; 4 Department of Pediatric Cardiothoracic Surgery, First Affiliated Hospital of Xinjiang Medical University, Urumqi, Xinjiang, China

**Keywords:** vasovagal syncope, heart rate variability, Holter, head-up tilt test, case-control study

## Abstract

**Background:**

Vasovagal syncope (VVS) is the most common type of reflex syncope. Although typically benign in its clinical course, VVS may lead to injury and reduced quality of life. Autonomic nervous system imbalance is considered the core pathophysiological mechanism of VVS. Heart rate variability (HRV), a noninvasive marker of autonomic regulation, may have practical value in identifying VVS and its subtypes; however, its predictive utility has not been fully elucidated.

**Methods:**

In this single-center retrospective case-control study, we included 415 patients with syncope symptoms who underwent both 24-hour Holter monitoring and a head-up tilt test (HUTT) between January 2021 and December 2024. Based on HUTT results, patients were classified into a VVS-positive group (*n* = 279) and a control group (*n* = 136). HRV parameters extracted from Holter recordings included 24 h average, maximum and minimum heart rates (HRs), standard deviation of NN intervals (SDNN), triangular index (TI), root mean square of successive differences (rMSSD), and the percentage of NN intervals differing by more than 50 ms (pNN50). Associations and predictive performance were assessed using logistic regression and receiver operating characteristic (ROC) analysis.

**Results:**

Multivariable logistic regression revealed that 24 h average HRs (OR: 0.935; 95% CI: 0.912–0.959; *P* < 0.001), 24 h maximum HRs (OR: 0.976; 95% CI: 0.964–0.989; *P* < 0.001), 24 h minimum HRs (OR: 0.947; 95% CI: 0.915–0.980; *P* = 0.002), TI (OR: 1.032; 95% CI: 1.009–1.056; *P* = 0.006), SDNN (OR: 1.029; 95% CI: 1.016–1.043; *P* < 0.001), rMSSD (OR: 1.023; 95% CI: 1.007–1.038; *P* = 0.004), and pNN50 (OR: 1.028; 95% CI: 1.006–1.051; *P* = 0.013) were independently associated with the occurrence of VVS. ROC analysis showed that 24 h average HRs (AUC: 0.688; 95% CI: 0.632–0.744), 24 h maximum HRs (AUC: 0.652; 95% CI: 0.594–0.709), and SDNN (AUC: 0.614; 95% CI: 0.557–0.672) exhibited moderate predictive ability for VVS.

**Conclusion:**

HRV parameters are associated with the occurrence of VVS. As a noninvasive and continuous physiological biomarker, HRV may aid in the clinical screening, risk stratification, and phenotypic classification of patients with suspected VVS.

## Background

Syncope is one of the common clinical symptoms and refers to a sudden and transient loss of consciousness caused by brief, reversible cerebral hypoperfusion, usually accompanied by postural collapse and followed by spontaneous recovery within minutes (1). Based on underlying pathophysiological mechanisms, syncope is generally classified into three major categories: reflex syncope, cardiac syncope, and orthostatic hypotension-related syncope (2). Vasovagal syncope (VVS) is the most common form of reflex syncope, accounting for approximately 30% to 40% of all syncope cases (3). VVS predominantly affects adolescents and young adults, and is characterized by sudden onset, variable symptoms, and a high recurrence rate (4). The pathogenesis of VVS is primarily attributed to autonomic dysregulation, manifested as abrupt withdrawal of sympathetic activity and/or exaggerated vagal response, leading to a concomitant decline in heart rates (HRs) and blood pressure and resulting in transient cerebral hypoperfusion (5). VVS episodes are often triggered by predisposing factors such as prolonged standing, emotional stress, or pain. These stimuli promote lower extremity venous pooling and reduced venous return, causing inadequate left ventricular filling and heightened sensitivity of ventricular wall mechanoreceptors (6). Although VVS typically follows a benign course, it may lead to fall-related injuries, traffic accidents, or other secondary harm during an episode, posing a potential public health and safety risk (7).

The head-up tilt test (HUTT) is currently the most widely used provocation method for assessing VVS in clinical settings. By simulating orthostatic hemodynamic stress observed in daily life, HUTT induces syncope or presyncope symptoms, thereby assisting in the differential diagnosis of underlying causes (8, 9). However, how to achieve noninvasive, dynamic, and early prediction of VVS episodes remains a pressing challenge in the field of autonomic function assessment (10). Heart rate variability (HRV), defined as the physiological fluctuation in consecutive RR intervals under sinus rhythm, reflects temporal changes in cardiac autonomic modulation and is considered a key marker of the autonomic nervous system's adaptability to internal and external stimuli (11). HRV is jointly regulated by sympathetic and parasympathetic branches of the autonomic nervous system and is influenced by respiratory rhythm, blood pressure regulation, emotional state, and metabolic status (12). Previous studies have demonstrated the prognostic value of HRV in patients with atrial fibrillation, heart failure, and myocardial infarction (13, 14). In patients with VVS, HRV has been shown to reflect baseline autonomic tone as well as pre-syncopal changes, indicating its potential as a predictive biomarker and interventional window (15, 16). Nevertheless, systematic comparisons of different HRV parameters in VVS remain limited, and their specificity across various clinical subtypes of VVS has not been clearly defined. Therefore, this study aims to analyze the characteristic patterns of HRV parameters in patients with VVS and to further evaluate their predictive value in both overall VVS and its distinct subtypes, providing a theoretical basis and quantitative tool to support individualized clinical management strategies.

## Methods

### Study design

This was a single-center retrospective case-control study. Hospitalized patients who underwent both 24 h Holter monitoring and HUTT in the Department of Cardiac Function, First Affiliated Hospital of Xinjiang Medical University, between January 2021 and December 2024 were consecutively enrolled. The study was conducted by the principles of the Declaration of Helsinki and was approved by the Human Ethics Committee of the First Affiliated Hospital of Xinjiang Medical University.

### Study population

Patients were included if they met all of the following criteria: (1) age ≥ 18 years; (2) a documented history of syncope triggered by prolonged standing, pain, or emotional stress; (3) completion of 24 h Holter monitoring during hospitalization; and (4) completion of a full HUTT after admission. Exclusion criteria were as follows: (1) presence of structural heart disease (e.g., valvular disease, cardiomyopathy, or congenital heart disease); (2) cardiogenic syncope (e.g., ventricular arrhythmias, atrioventricular block, or a family history of sudden cardiac death); (3) neurogenic syncope (e.g., epilepsy or psychogenic pseudosyncope); (4) current diagnosis of psychiatric disorders or use of antidepressants, antiepileptics, or other neuroactive medications; and (5) Holter monitoring duration less than 6 h.

### Clinical data collection

Demographic and clinical data were retrospectively collected from the electronic medical records, including age, sex, body mass index (BMI), history of hypertension, diabetes mellitus, and cardiovascular disease (CVD) at the time of hospital admission. Laboratory tests included hemoglobin, serum potassium (K^+^), glycated hemoglobin (HbA1c), N-terminal pro-brain natriuretic peptide (NT-proBNP), and interleukin-6 (IL-6). A history of hypertension was defined based on patient self-report, ambulatory blood pressure monitoring results, or current use of antihypertensive medications. Cardiovascular diseases included coronary artery disease, heart failure, angina pectoris, myocardial infarction, or stroke. Diabetes mellitus was defined by one or more of the following criteria: self-reported history, HbA1c level >6.5%, fasting plasma glucose ≥7.0 mmol/L, or use of glucose-lowering medications or insulin.

### 24-hour Holter monitoring

Within 24 h of admission, all patients underwent 24 h Holter monitoring using the MedEx MECG-200 device (Beijing, China). Recordings were acquired at a sampling frequency of 250 Hz, with patients instructed to maintain their usual daily activities during monitoring. Following completion of the 24 h recording, the device was retrieved. MedEx analysis software automatically detected artifacts, premature ectopic beats, and segments with noise contamination, which were then manually reviewed and confirmed by two physicians with intermediate or higher titles. Segments requiring editing (RR interval <5%) or with noise contamination >10% were excluded from each record. Only normal heartbeat intervals (NN intervals) were used to calculate heart rate variability indices. The following parameters were extracted: 24-hour maximum HRs, minimum HRs, and average HRs; standard deviation of normal-to-normal RR intervals (SDNN); triangular index (TI); root mean square of successive differences (rMSSD); percentage of successive RR intervals differing by more than 50 ms (pNN50); low-frequency power (LF); high-frequency power (HF); and the ratio of low-frequency to high-frequency power (LF: HF).

### Head-up tilt test

All patients in this study underwent a standardized HUTT with continuous electrocardiographic and noninvasive blood pressure monitoring. The procedure was conducted in a quiet, temperature-controlled examination room, following the protocol recommended by the 2018 Chinese Expert Consensus on the Diagnosis and Treatment of Syncope (17). All participants were required to fast for at least 4 h and discontinue any medications that might affect autonomic function for at least 48 h before testing. The test protocol was as follows: patients rested in the supine position on a tilt table for 10 min while baseline ECG and blood pressure were recorded. The table was then tilted gradually to a 70° angle. During the tilt phase, patients were continuously monitored for presyncope symptoms such as dizziness, nausea, pallor, or sweating, as well as for typical autonomic responses such as sudden drops in heart rate or blood pressure. The passive phase lasted for up to 40 min. For patients without a positive response during the passive phase, 5 μg/kg of sublingual nitroglycerin or an equivalent dose of isoproterenol was administered. The pharmacological phase continued for an additional 20 min. If a positive response occurred, the test was immediately terminated; if no response occurred within 20 min, the test was considered negative. For patients with a positive response, the type of reaction was recorded and classified into one of the three subtypes of VVS based on heart rate and blood pressure patterns: mixed type, cardioinhibitory type, or vasodepressor type.

### Statistical analysis

All statistical analyses were performed using R software (version 4.4.2). Based on the HUTT results, participants were divided into the VVS-positive group and the control group. Baseline characteristics, HRV parameters, and HUTT outcomes were compared between groups. Continuous variables with non-normal distributions were expressed as median and interquartile range (25th and 75th percentiles), and differences between groups were assessed using the Mann–Whitney U test. Categorical variables were presented as frequencies (percentages) and compared using the chi-square test. To compare HRV parameters among different VVS subtypes, the Kruskal–Wallis test was employed. Spearman correlation analysis was used to examine the associations between various HRV parameters. Logistic regression analysis was used to explore the independent associations between HRV and the presence of VVS and its subtypes. The predictive ability of each HRV parameter for identifying VVS and its subtypes was assessed using receiver operating characteristic (ROC) curve analysis. A two-tailed *P*-value < 0.05 was considered statistically significant for all analyses.

## Results

### Baseline characteristics of participants

A total of 415 patients with syncope symptoms were enrolled in this study. The median age was 52 years, with 212 (51.1%) males and 203 (48.9%) females. Based on the results of the HUTT, 279 patients were classified into the positive group and 136 into the control group. There were no significant differences between the two groups in terms of sex, prevalence of diabetes mellitus, CVD, hemoglobin levels, serum potassium, or HbA1c levels (*P* > 0.05). However, the positive group had significantly higher age, prevalence of hypertension, NT-proBNP levels, and IL-6 levels, while BMI was significantly lower compared to the control group (*P* < 0.05) (Table 1). In the HUTT, patients in the positive group exhibited significantly lower values in tilt duration, supine heart rate, minimum systolic blood pressure, minimum diastolic blood pressure, and minimum heart rate (*P* < 0.05). In contrast, the maximum heart rate change during the test was significantly greater in the positive group compared to the control group (*P* < 0.05). There were no significant differences in supine systolic or diastolic blood pressure between the two groups (*P* > 0.05) (Table 2).

**Table 1:** General clinical information of participants.

Characteristics	Total (*n* = 415)	Control group (*n* = 136)	Positive group (*n* = 279)	*χ*^2^/*U*	*P*-value
Sex					
Male	212 (51.1)	75 (55.1)	137 (49.1)	1.336	0.248
Female	203 (48.9)	61 (44.9)	142 (50.9)		
Diabetes	26 (6.3)	7 (5.1)	19 (6.8)	0.431	0.512
CVD	71 (17.1)	17 (12.5)	54 (19.4)	3.029	0.082
Hypertension	113 (27.2)	26 (19.1)	87 (31.2)	6.717	0.010
Age, years	52.0 (44.0,62.0)	49.0 (43.0,56.0)	56.0 (44.0,64.0)	−3.567	<0.001
BMI, kg/m^2^	24.7 (22.6,27.2)	25.9 (23.6,28.1)	24.2 (22.2,26.2)	5.299	<0.001
Hemoglobin, g/L	134.0 (125.0,144.0)	132.0 (125.0,143.0)	135.0 (125.0,145.0)	−0.436	0.663
K+, mmolL	3.79 (3.60,3.99)	3.85 (3.63,4.00)	3.78 (3.58,3.99)	1.431	0.152
HbA1C, %	5.59 (5.40,5.85)	5.56 (5.40,5.76)	5.60 (5.40,5.90)	−1.772	0.076
NT-proBNP, pg/mL	43.0 (23.0,72.7)	29.9 (20.9,52.4)	51.1 (27.2,91.3)	−4.837	<0.001
IL-6, pg/mL	2.57 (1.71,5.15)	1.94 (1.50,3.40)	3.00 (1.94,6.14)	−4.856	<0.001

BMI, body mass index; HbA1C, glycated hemoglobin; NT-proBNP, N-terminal pro-brain natriuretic peptide; IL-6, interleukin-6; CVD, cardiovascular disease.

**Table 2:** HUTT records of patients in the control group and positive group.

Characteristics	Control group	Positive group	*U*	*P*-value
Tilt duration, min	40.0 (40.0,40.0)	26.0 (23.0,29.0)	16.483	<0.001
Supine period SBP, mmHg	121.0 (112.0,134.0)	123.0 (114.0,134.0)	−1.052	0.293
Supine period DBP, mmHg	75.0 (66.0,82.0)	77.0 (70.0,84.0)	−1.835	0.066
Supine period HRs, bpm	74.0 (67.0,88.0)	68.0 (62.0,77.0)	4.4	<0.001
Minimum SBP, mmHg	103.0 (95.0,111.0)	76.0 (68.0,80.0)	15.163	<0.001
Lowest DBP, mmHg	70.0 (65.0,78.0)	47.0 (42.0,52.0)	15.346	<0.001
Maximum HRs change, bpm	0.0 (0.0,5.0)	16.0 (8.0,23.0)	−11.906	<0.001
Minimum HRs, bpm	71.0 (64.0,79.0)	54.0 (44.0,61.0)	11.808	<0.001

SBP, systolic blood pressure; DBP, diastolic blood pressure; HRs, heart rates.

### Relationship between HRV and VVS

**Figure 1:**
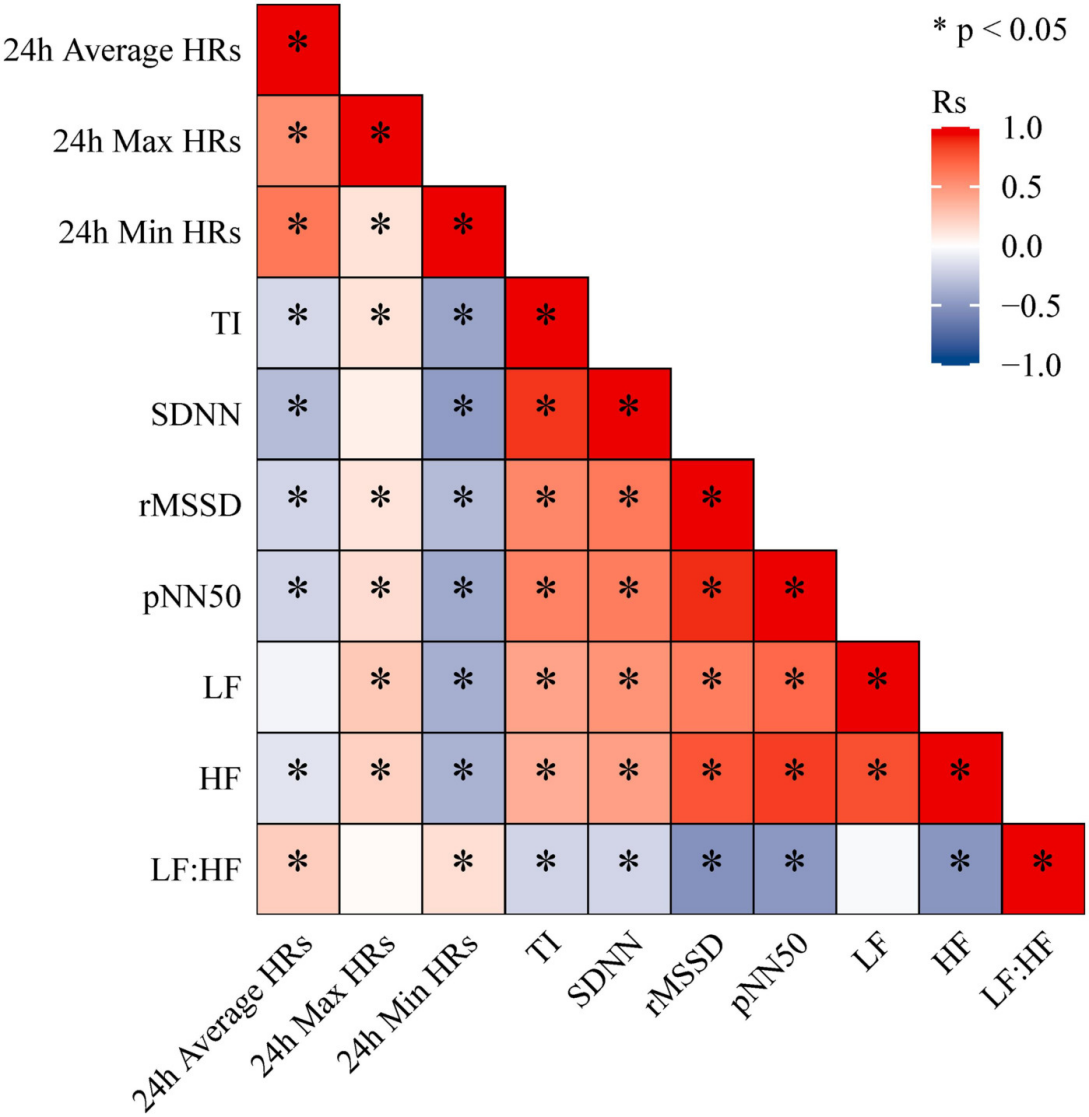
Spearman correlation heatmap between 24-hour holter monitoring indicators.

Spearman correlation analysis demonstrated that heart rate parameters and HRV indices were moderately associated. Specifically, 24 h Average HRs and 24 h Min HRs were positively correlated with HRV measures, whereas 24 h Max HRs showed negative correlations. In addition, TI, SDNN, rMSSD, pNN50, LF, and HF were all positively correlated with one another (Figure 1). This study demonstrated that patients in the positive group had significantly lower 24 h Average HRs, 24 h Max HRs, 24 h Min HRs, whereas the levels of TI, SDNN, rMSSD, and pNN50 were significantly higher compared to the control group (*P* < 0.05) (Table 3). Among the 279 patients with a positive HUTT, 23 were classified as cardioinhibitory type, 124 as mixed type, and 132 as vasodepressor type. Further analysis revealed significant differences among the three VVS subtypes in terms of 24 h Max HR, 24 h Min HR, TI, SDNN, rMSSD, pNN50, LF, and HF (*P* < 0.05) (Table 4).

**Table 3:** HRV parameters of patients in the control group and positive group.

Characteristics	Total	Control group	Positive group	*U*	*P*-value
24 h Average HRs	73.0 (68.0,79.0)	77.0 (72.0,85.0)	72.0 (66.0,77.0)	6.220	<0.001
24 h Max HRs	126.0 (116.0,140.0)	135.0 (120.0,149.0)	123.0 (114.0,135.0)	5.024	<0.001
24 h Min HRs	50.0 (46.0,54.0)	51.0 (46.0,55.0)	49.0 (45.0,53.0)	2.123	0.034
TI	32.6 (27.2,40.3)	32.9 (26.2,38.7)	32.5 (28.2,40.9)	−1.548	0.122
SDNN	74.6 (64.8,92.3)	70.5 (60.3,85.8)	77.2 (66.1,93.6)	−3.787	<0.001
rMSSD	30.7 (23.3,42.6)	27.9 (20.0,39.4)	31.4 (24.6,45.6)	−3.035	0.002
pNN50	7.7 (3.3,16.4)	6.5 (2.1,16.9)	7.9 (3.9,16.3)	−2.133	0.033
LF	512.0 (346.1,774.3)	502.8 (305.3,780.3)	518.6 (359.3,769.0)	−0.956	0.339
HF	219.0 (138.8,445.6)	218.1 (113.5,516.4)	219.9 (146.2,432.6)	−0.892	0.372
LF: HF	2.8 (2.1,3.7)	3.0 (2.3,3.8)	2.7 (2.1,3.6)	1.193	0.233

**Table 4:** HRV parameters of patients with different types of VVS.

Characteristics	Vasodepressor (*n* = 132)	Cardioinhibitory (*n* = 23)	Mixed (*n* = 124)	*U*	*P*-value
24 h Average HRs	72.0 (66.0,75.0)	70.0 (67.0,75.0)	72.0 (66.0,79.0)	2.229	0.328
24 h Max HRs	121.0 (111.0,134.0)	124.0 (116.0,133.0)	127.0 (117.0,140.0)	9.961	0.007
24 h Min HRs	50.0 (47.0,53.0)	46.0 (40.0,50.0)	49.0 (45.0,54.0)	7.327	0.026
TI	31.1 (26.7,37.7)	41.1 (35.2,45.5)	34.1 (30.0,41.2)	12.822	0.002
SDNN	75.4 (65.0,90.7)	91.1 (75.8,102.3)	77.3 (67.0,94.6)	6.621	0.036
rMSSD	28.8 (23.4,39.6)	45.6 (32.8,49.5)	34.2 (25.1,45.0)	13.947	<0.001
pNN50	6.1 (3.2,12.7)	14.0 (9.2,25.9)	9.4 (4.6,19.3)	19.908	<0.001
LF	462.6 (311.5,657.2)	750.7 (470.3,829.3)	544.6 (375.7,878.5)	13.049	0.001
HF	184.0 (131.1,333.4)	289.2 (219.9,573.3)	257.6 (158.0,468.5)	15.357	<0.001
LF: HF	3.0 (2.2,3.9)	2.7 (2.1,3.2)	2.5 (2.0,3.4)	4.389	0.111

Univariate logistic regression analysis indicated that 24 h Average HRs, 24 h Max HRs, 24 h Min HRs, TI, SDNN, and rMSSD were significantly associated with the occurrence of VVS. After adjusting for age, BMI, history of hypertension, NT-proBNP, and IL-6, multivariate logistic regression analysis showed that 24 h Average HRs (OR: 0.935; 95% CI: 0.912–0.959; *P* < 0.001), 24 h Max HRs (OR: 0.976; 95% CI: 0.964–0.989; *P* < 0.001), 24 h Min HRs (OR: 0.947; 95% CI: 0.915–0.980; *P* = 0.002), TI (OR: 1.032; 95% CI: 1.009–1.056; *P* = 0.006), SDNN (OR: 1.029; 95% CI: 1.016–1.043; *P* < 0.001), rMSSD (OR: 1.023; 95% CI: 1.007–1.038; *P* = 0.004), and pNN50 (OR: 1.028; 95% CI: 1.006–1.051; *P* = 0.013) were independently associated with the risk of VVS (Table 5).

**Table 5:** Logistic regression of HRV and occurrence of VVS in participants.

Characteristics	OR(95% CI)	*P*-value	OR(95% CI)-adjusted	*P*-value-adjusted
24 h Average HR	0.929 (0.907,0.951)	<0.001	0.935 (0.912,0.959)	<0.001
24 h Max HR	0.972 (0.96,0.983)	<0.001	0.976 (0.964,0.989)	<0.001
24 h Min HR	0.955 (0.926,0.986)	0.004	0.947 (0.915,0.980)	0.002
TI	1.023 (1.006,1.04)	0.008	1.032 (1.009,1.056)	0.006
SDNN	1.022 (1.012,1.032)	<0.001	1.029 (1.016,1.043)	<0.001
rMSSD	1.016 (1.003,1.029)	0.014	1.023 (1.007,1.038)	0.004
pNN50	1.014 (0.996,1.031)	0.129	1.028 (1.006,1.051)	0.013
LF	1.000 (1.000,1.000)	0.901	1.000 (1.000,1.000)	0.942
HF	1.000 (1.000,1.000)	0.911	1.000 (1.000,1.000)	0.981
LF: HF	0.905 (0.779,1.051)	0.192	0.918 (0.778,1.083)	0.309

Further logistic regression analyses by VVS subtype showed that 24 h Average HRs, 24 h Max HRs, 24 h Min HRs, TI, and SDNN were independently associated with both vasodepressor and cardioinhibitory types (Supplementary Table S1). pNN50 was also independently associated with the cardioinhibitory type (Supplementary Table S2). In addition, 24 h Average HRs, 24 h Min HRs, TI, SDNN, rMSSD, and pNN50 were independently associated with the mixed type of VVS (Supplementary Table S3).

### Predictive value of HRV for VVS

**Figure 2:**
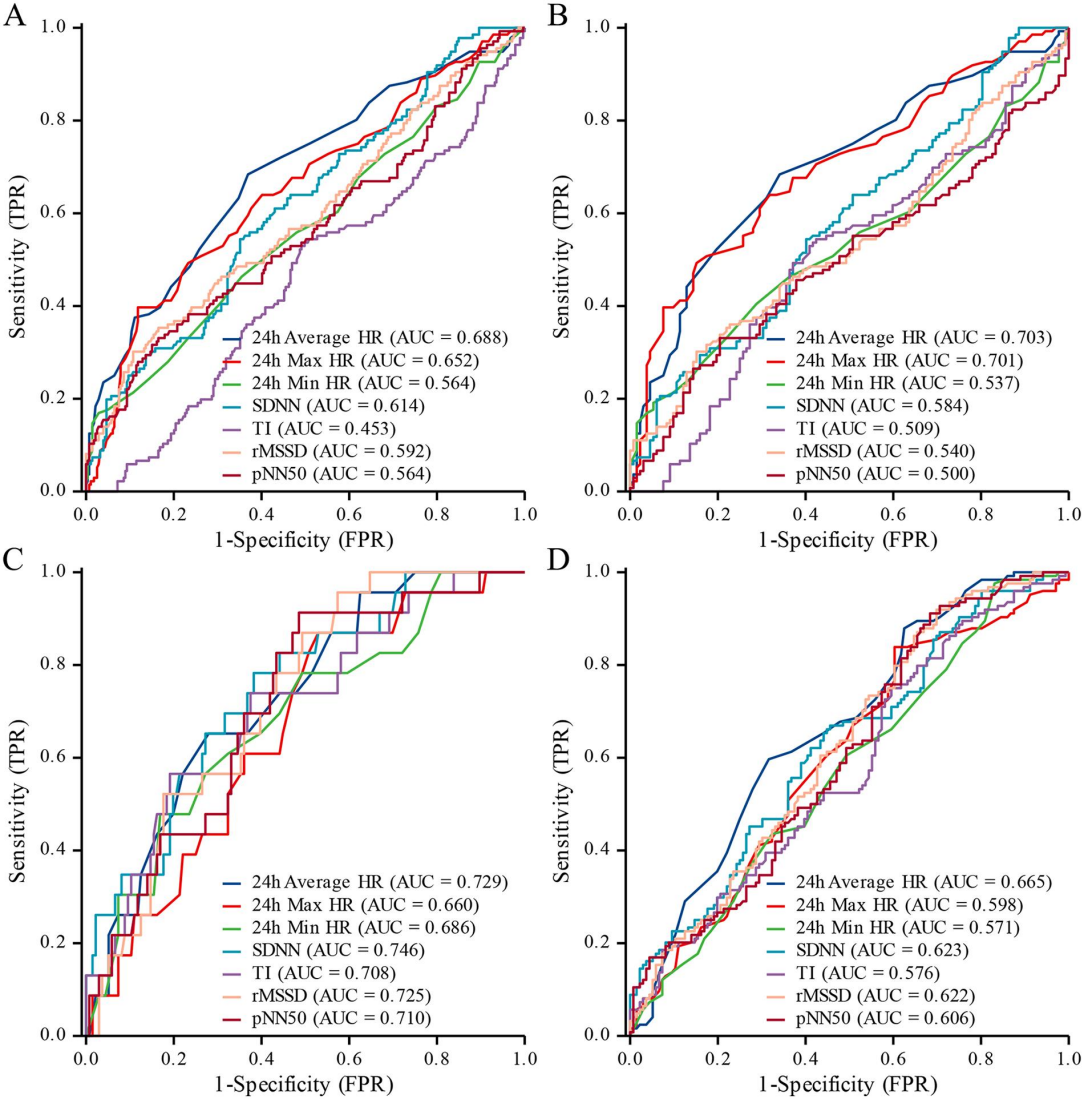
ROC curves for HRV prediction of VVS and subtypes. **(A)** All types; **(B)** vasodepressor type; **(C)** cardioinhibitory type; **(D)** mixed type.

This study assessed the predictive performance of HRV parameters for VVS and its subtypes using ROC curve analysis (Table 6). The results showed that 24 h Average HRs (AUC: 0.688; 95% CI: 0.632–0.744), 24 h Max HRs (AUC: 0.652; 95% CI: 0.594–0.709), and SDNN (AUC: 0.614; 95% CI: 0.557–0.672) had moderate ability to predict the presence of VVS (Figure 2A). For vasodepressor-type VVS, both 24 h Average HRs (AUC: 0.703; 95% CI: 0.640–0.765) and 24 h Max HRs (AUC: 0.701; 95% CI: 0.638–0.763) demonstrated good predictive performance. In contrast, SDNN (AUC: 0.584; 95% CI: 0.516–0.652) and rMSSD (AUC: 0.540; 95% CI: 0.471–0.610) showed relatively lower predictive value (Figure 2B). Regarding the prediction of cardioinhibitory-type VVS, SDNN (AUC: 0.746; 95% CI: 0.642–0.850), 24 h Average HRs (AUC: 0.729; 95% CI: 0.627–0.832), and rMSSD (AUC: 0.725; 95% CI: 0.631–0.820) all exhibited good predictive accuracy. Additionally, pNN50 (AUC: 0.710; 95% CI: 0.605–0.815) and TI (AUC: 0.708; 95% CI: 0.593–0.823) also showed strong discriminative ability (Figure 2C). For the mixed-type VVS, 24 h Average HRs (AUC: 0.665; 95% CI: 0.599–0.730), SDNN (AUC: 0.623; 95% CI: 0.555–0.690), rMSSD (AUC: 0.622; 95% CI: 0.554–0.689), and pNN50 (AUC: 0.606; 95% CI: 0.537–0.674) were found to have favorable predictive performance (Figure 2D). Detailed ROC parameters for predicting specific VVS subtypes are presented in the (Supplementary Tables S4–S6).

**Table 6:** ROC analysis of HRV prediction of VVS.

Variable	AUC (95% CI)	Cut-off value	Sensitivity	Specificity	Youden index
24 h Average HR	0.688 (0.632–0.744)	73.5	68.4%	63.1%	0.315
24 h Max HR	0.652 (0.594–0.709)	143.5	39.7%	88.2%	0.279
24 h Min HR	0.564 (0.503–0.625)	59.5	16.9%	97.1%	0.140
SDNN	0.614 (0.557–0.672)	70.9	54.4%	64.9%	0.193
TI	0.453 (0.394–0.512)	32.5	53.7%	50.2%	0.039
rMSSD	0.592 (0.531–0.652)	20.9	30.1%	89.2%	0.194
pNN50	0.564 (0.503–0.626)	2.9	33.1%	83.5%	0.166

AUC, area under the receiver operating characteristic curve.

## Discussion

The findings of this study suggest that HRV parameters hold certain clinical value in distinguishing individuals with VVS from those without, as well as in differentiating among VVS subtypes. Specifically, 24 h Average HRs, 24 h Max HRs, 24 h Min HRs, TI, SDNN, rMSSD, and pNN50 were all associated with VVS and demonstrated a degree of predictive ability.

The core pathophysiological mechanism of VVS involves a synergistic effect between enhanced vagal activity and suppressed sympathetic activity (18). In the present study, we observed that 24 h average, maximum, and minimum HRs were negatively correlated with the presence of VVS, whereas TI, SDNN, rMSSD, and pNN50 were positively correlated. The 24 h average, maximum, and minimum HRs are fundamental statistical measures reflecting the overall, peak, and trough heart rate levels over a 24 h period, respectively, and are primarily influenced by sympathetic nervous activity (19). An elevated heart rate typically reflects enhanced sympathetic activation or reduced parasympathetic tone, while a reduced heart rate is often indicative of increased vagal activity or decreased sympathetic drive. SDNN, TI, rMSSD, and pNN50 are all time-domain measures of HRV, which collectively reflect the integrated state of autonomic nervous system modulation. An upward trend in these parameters suggests increased autonomic fluctuations and stronger vagal influence in daily physiological rhythms (20). Notably, rMSSD and pNN50 are highly sensitive to parasympathetic activity; elevated values indicate vagal hyperexcitability even during non-syncope periods, which may provide a physiological basis for the rapid onset of vagal reflexes in response to specific triggers (21). The increase in SDNN further implies heightened overall HRV variability across 24 h, representing a state of autonomic instability between sympathetic and parasympathetic systems. This intrinsic feature may underlie syncope susceptibility. HF power has traditionally been regarded as the most specific frequency-domain marker of cardiac parasympathetic regulation. However, HF power is highly sensitive to respiratory patterns—such as respiratory irregularity and respiratory rate variability—neither of which were standardized or recorded in this retrospective cohort study. Respiratory variability during daily activities may introduce significant noise into HF power measurements, weakening its association with vagal tone (22). In contrast, rMSSD and pNN50 are less affected by respiratory variability and are considered more robust markers of short-term vagal regulation in free-living conditions (23). Twenty-four-hour HF power reflects the cumulative effects of sleep-related respiratory rhythms and daytime behavioral factors, potentially diminishing its sensitivity in distinguishing subtle differences between vagal dysfunction subtypes. In contrast, time-domain metrics capture inter-beat parasympathetic fluctuations and are more likely to reflect increased intrinsic vagal excitability associated with cardiac suppression responses.

There are significant differences in autonomic reflex characteristics among the three clinical subtypes of VVS (24). In this study, patients with cardioinhibitory VVS generally exhibited higher HRV levels, with parameters such as SDNN, rMSSD, and pNN50 demonstrating strong predictive performance. The hallmark of the cardioinhibitory subtype is a markedly enhanced vagal response, manifesting as sinus bradycardia, atrioventricular conduction delay, or even transient cardiac arrest (25). The underlying mechanism is attributed to the hypersensitivity of cardiac mechanoreceptors to mechanical stretch. Under triggering conditions, these receptors transmit strong inhibitory signals via the vagus nerve to the central nervous system, thereby activating cardioinhibitory reflexes. This results in sinoatrial node suppression, abrupt reduction in cardiac output, and subsequent loss of consciousness (26, 27). Elevated HRV in this subtype suggests a predominance of parasympathetic tone even during interictal periods, indicating a physiological predisposition to vagal hyperactivity that can rapidly precipitate syncope upon provocation (28).

The vasodepressor subtype is primarily triggered by sudden sympathetic withdrawal, leading to peripheral vasodilation. Episodes are often characterized by a rapid drop in blood pressure with minimal changes in heart rate (29). This reflects impaired regulation of arterial smooth muscle tone. In the presence of postural stress or abnormal blood volume distribution, the sympathetic-mediated vascular tone fails to compensate, resulting in a sharp decline in effective circulating blood volume (30). Because heart rate fluctuations are relatively mild, HRV variability in this subtype is generally less pronounced compared to the cardioinhibitory type (31). Our findings showed that the predictive performance of most HRV parameters in this subtype was also relatively limited. While 24 h average and maximum heart rates retained some discriminatory power, possibly reflecting a baseline reduction in sympathetic tone, other indices such as SDNN, rMSSD, and pNN50 exhibited low predictive value. This is consistent with clinical observations that vasodepressor VVS is primarily characterized by hypotension without prominent bradycardia (32).

The mixed type of VVS encompasses features of both the cardioinhibitory and vasodepressor subtypes. During syncopal episodes, patients experience both a reduction in heart rate and a significant drop in blood pressure (33). This dual response reflects a global imbalance in autonomic regulation, characterized by a loss of coordinated sympathetic and parasympathetic control, resulting in simultaneous instability of both heart rate and blood pressure (34). As the most common subtype observed in clinical settings, mixed-type VVS typically exhibits intermediate HRV characteristics, with its predictive capacity showing variability depending on individual autonomic profiles. In the present study, HRV parameters in mixed-type patients were generally between those of the other two subtypes. While rMSSD, pNN50, and SDNN exhibited an upward trend, their predictive performance was slightly lower than that observed in the cardioinhibitory group. To date, there is a relative paucity of literature addressing HRV parameter differences across VVS subtypes. Many previous studies have not stratified VVS into distinct clinical forms or have solely focused on differences between tilt-positive and tilt-negative individuals, thereby overlooking the unique autonomic patterns associated with different pathophysiological mechanisms (35, 36). Some recent efforts have attempted to integrate HRV with blood pressure variability (BPV) or dynamic indices derived from tilt-table testing to enhance predictive accuracy. For example, Virag et al. used RR intervals and SBP trends during HUTT to predict impending syncope (37). However, the clinical utility of such models is limited by their complexity and dependence on operator expertise. In contrast, the findings of this study suggest that HRV serves as a valuable adjunct diagnostic tool. Its methodological simplicity, capacity for continuous noninvasive monitoring, and compatibility with wearable medical devices make it a highly promising approach for identifying high-risk individuals for VVS in real-world and outpatient settings (38). This study focused on 24 h HRV reflecting autonomic nervous system status across a full circadian rhythm cycle. However, short-term HRV based on 5 min or ultra-short recordings can capture more immediate parasympathetic fluctuations, potentially offering complementary diagnostic information (39). Wearable or low-cost photoplethysmography (PPG) devices can perform short-term HRV analysis. Future studies may consider integrating low-cost HRV monitoring solutions to achieve continuous autonomic monitoring in real-world settings (40).

Recent evidence indicates that heart rate variability biofeedback (HRV-BF) serves as an evidence-based non-pharmacological intervention aimed at enhancing autonomic nervous system regulation by self-modulating cardiopulmonary rhythms (41). HRV-BF typically guides individuals to breathe at a personalized resonance frequency (approximately 0.1 Hz), where respiratory sinus arrhythmia reaches its maximum expression and baroreflex sensitivity is heightened. This synergistic activation of the cardiac vagal pathway induces more stable oscillatory patterns in heart rate and blood pressure, thereby promoting sympathetic-parasympathetic equilibrium (42). Clinical studies confirm that regular HRV-BF training enhances vagal tone, improves baroreflex function, and reduces autonomic hypersensitivity, indicating its potential efficacy for autonomic dysregulation disorders (43). For patients with cardiac-suppressed VVS, such methods may help inhibit excessive vagal impulses; whereas those with vascular-suppressed VVS may benefit from enhanced sympathetic-baroreflex coupling. Therefore, incorporating subtype-specific breathing guidance into clinical management not only complements HRV diagnostic assessment but also reduces syncope susceptibility through personalized autonomic regulation.

## Limitations

This study has several limitations. First, as a retrospective analysis based on previously collected medical records, certain potential confounding factors—such as sleep status and emotional disturbances—may not have been adequately controlled. Second, the study population was derived from a single-center cohort consisting of patients with syncope symptoms who underwent both 24 h Holter monitoring and HUTT, which may limit the generalizability of the findings. Therefore, the observed characteristics of VVS may not fully reflect those in the general population or in asymptomatic individuals. Third, this study evaluated the discriminatory ability of HRV parameters for different VVS subtypes through ROC analysis. However, the sample size in the cardiac suppression subtype group was relatively small, necessitating larger-scale studies to validate the role of HRV in this subtype.

## Conclusion

In conclusion, 24 h Average HRs, 24 h Max HRs, 24 h Min HRs, TI, SDNN, rMSSD, and pNN50 were all found to be significantly associated with VVS. As a noninvasive, objective, and dynamic physiological measure, HRV reflects the regulatory capacity of the autonomic nervous system and may serve as a valuable tool in the clinical screening, risk prediction, and subtype classification of VVS.

## Data Availability

The raw data supporting the conclusions of this article will be made available, upon reasonable request to the corresponding author.
