# Supplementary material for: Evaluation of the clinical value of heart rate variability in predicting vasovagal syncope
Source: Front Cardiovasc Med. 2026 Jan 21;12:1684990. doi: 10.3389/fcvm.2025.1684990 (PMC12869434; doi:10.3389/fcvm.2025.1684990)
Supplement: Supplementary file 1 [file Supplementaryfile1.docx]

Table S1: Holter parameters and logistic regression of vasodepressor VVS

| **Characteristics** | **OR(95% CI)** | **P-value** | **OR(95% CI)-adjusted** | **P-value-adjusted** |
| --- | --- | --- | --- | --- |
| 24h Average HR | 0.93(0.904,0.956) | <0.001 | 0.943(0.915,0.972) | <0.001 |
| 24h Max HR | 0.958(0.943,0.973) | <0.001 | 0.967(0.950,0.985) | <0.001 |
| 24h Min HR | 0.971(0.936,1.007) | 0.11 | 0.959(0.920,1.000) | 0.048 |
| TI | 1.015(0.997,1.032) | 0.101 | 1.028(1.002,1.055) | 0.035 |
| SDNN | 1.018(1.007,1.029) | 0.001 | 1.027(1.012,1.042) | <0.001 |
| rMSSD | 1.004(0.988,1.019) | 0.645 | 1.012(0.994,1.031) | 0.206 |
| pNN50 | 0.986(0.963,1.009) | 0.235 | 1.007(0.978,1.037) | 0.655 |
| LF | 1(0.999,1.0) | 0.125 | 1(1.000,1.000) | 0.646 |
| HF | 0.999(0.999,1.0) | 0.044 | 1(0.999,1.000) | 0.326 |
| LF: HF | 0.984(0.828,1.17) | 0.856 | 1.017(0.834,1.239) | 0.871 |
| Mean QTc interval | 0.996(0.986,1.005) | 0.348 | 0.993(0.982,1.004) | 0.209 |
| QTcD | 0.998(0.996,1.0) | 0.02 | 0.999(0.997,1.001) | 0.309 |

Note: “Adjusted” refers to adjustment for age, BMI, hypertension, NT-proBNP, and IL-6 as covariates.

Table S2: Holter parameters and logistic regression of cardioinhibitory VVS

| **Characteristics** | **OR(95% CI)** | **P-value** | **OR(95% CI)-adjusted** | **P-value-adjusted** |
| --- | --- | --- | --- | --- |
| 24h Average HR | 0.919(0.873,0.967) | 0.001 | 0.905(0.854,0.958) | 0.001 |
| 24h Max HR | 0.968(0.943,0.994) | 0.017 | 0.953(0.921,0.986) | 0.005 |
| 24h Min HR | 0.903(0.842,0.968) | 0.004 | 0.898(0.833,0.969) | 0.005 |
| TI | 1.062(1.026,1.099) | 0.001 | 1.074(1.030,1.120) | 0.001 |
| SDNN | 1.043(1.021,1.065) | <0.001 | 1.048(1.022,1.075) | <0.001 |
| rMSSD | 1.036(1.013,1.061) | 0.002 | 1.042(1.015,1.069) | 0.002 |
| pNN50 | 1.05(1.016,1.085) | 0.004 | 1.057(1.016,1.100) | 0.006 |
| LF | 1(1.0,1.0) | 0.922 | 1(1.000,1.000) | 0.996 |
| HF | 1(1.0,1.0) | 0.84 | 1(1.000,1.000) | 0.983 |
| LF: HF | 0.773(0.53,1.128) | 0.182 | 0.708(0.464,1.081) | 0.11 |
| Mean QTc interval | 0.997(0.982,1.011) | 0.636 | 0.993(0.977,1.010) | 0.419 |
| QTcD | 0.997(0.993,1.001) | 0.114 | 0.998(0.994,1.002) | 0.327 |

Note: “Adjusted” refers to adjustment for age, BMI, hypertension, NT-proBNP, and IL-6 as covariates.

Table S2: Holter parameters and logistic regression of mixed VVS

| **Characteristics** | **OR(95% CI)** | **P-value** | **OR(95% CI)-adjusted** | **P-value-adjusted** |
| --- | --- | --- | --- | --- |
| 24h Average HR | 0.937(0.911,0.963) | <0.001 | 0.943(0.915,0.971) | <0.001 |
| 24h Max HR | 0.983(0.971,0.996) | 0.013 | 0.989(0.974,1.003) | 0.127 |
| 24h Min HR | 0.958(0.926,0.992) | 0.017 | 0.956(0.920,0.993) | 0.020 |
| TI | 1.029(1.008,1.051) | 0.007 | 1.034(1.008,1.061) | 0.009 |
| SDNN | 1.023(1.011,1.036) | <0.001 | 1.027(1.013,1.042) | <0.001 |
| rMSSD | 1.021(1.007,1.035) | 0.004 | 1.026(1.010,1.043) | 0.002 |
| pNN50 | 1.026(1.007,1.046) | 0.008 | 1.034(1.010,1.058) | 0.006 |
| LF | 1(1.0,1.0) | 0.499 | 1(1.000,1.000) | 0.736 |
| HF | 1(1.0,1.0) | 0.484 | 1(1.000,1.000) | 0.642 |
| LF: HF | 0.852(0.709,1.024) | 0.088 | 0.854(0.696,1.048) | 0.131 |
| Mean QTc interval | 0.994(0.985,1.003) | 0.210 | 0.995(0.985,1.004) | 0.263 |
| QTcD | 1(0.999,1.001) | 0.993 | 1(0.999,1.001) | 0.961 |

Note: “Adjusted” refers to adjustment for age, BMI, hypertension, NT-proBNP, and IL-6 as covariates.

Table S4: ROC Analysis of HRV for predicting vasoinhibitory VVS

| Variable | AUC (95% CI) | Cut-off value | Sensitivity | Specificity | Youden Index |
| --- | --- | --- | --- | --- | --- |
| 24h Average HR | 0.703(0.640 – 0.765) | 73.5 | 68.4% | 65.9% | 0.343 |
| 24h Max HR | 0.701(0.638 – 0.763) | 135.5 | 49.3% | 84.8% | 0.341 |
| 24h Min HR | 0.537(0.467 – 0.607) | 57.5 | 19.1% | 94.7% | 0.138 |
| SDNN | 0.584(0.516 – 0.652) | 70.9 | 54.4% | 59.8% | 0.143 |
| TI | 0.509(0.439 – 0.579) | 33.1 | 49.3% | 62.9% | 0.121 |
| rMSSD | 0.54(0.471 – 0.610) | 20.9 | 30.1% | 84.8% | 0.150 |
| pNN50 | 0.5(0.429 – 0.570) | 13.8 | 33.1% | 79.5% | 0.126 |

Table S5: ROC Analysis of HRV for predicting cardioinhibitory VVS

| Variable | AUC (95% CI) | Cut-off value | Sensitivity | Specificity | Youden Index |
| --- | --- | --- | --- | --- | --- |
| 24h Average HR | 0.729(0.627 – 0.832) | 72.5 | 65.2% | 72.1% | 0.373 |
| 24h Max HR | 0.66(0.550 – 0.770) | 136.5 | 87.0% | 47.1% | 0.340 |
| 24h Min HR | 0.686(0.567 – 0.804) | 43.5 | 47.8% | 83.1% | 0.309 |
| SDNN | 0.746(0.642 – 0.850) | 75.2 | 78.3% | 61.8% | 0.400 |
| TI | 0.708(0.593 – 0.823) | 40.7 | 56.5% | 80.9% | 0.374 |
| rMSSD | 0.725(0.631 – 0.820) | 25.4 | 95.7% | 42.6% | 0.383 |
| pNN50 | 0.71(0.605 – 0.815) | 7.0 | 91.3% | 51.5% | 0.428 |

Table S6: ROC Analysis of HRV for predicting mixed VVS

| Variable | AUC (95% CI) | Cut-off value | Sensitivity | Specificity | Youden Index |
| --- | --- | --- | --- | --- | --- |
| 24h Average HR | 0.665(0.599 – 0.730) | 73.5 | 59.7% | 68.4% | 0.281 |
| 24h Max HR | 0.598(0.529 – 0.667) | 143.5 | 83.9% | 39.7% | 0.236 |
| 24h Min HR | 0.571(0.501 – 0.640) | 59.5 | 97.6% | 16.9% | 0.145 |
| SDNN | 0.623(0.555 – 0.690) | 71.0 | 66.9% | 54.4% | 0.213 |
| TI | 0.576(0.507 – 0.646) | 29.9 | 75.0% | 40.4% | 0.154 |
| rMSSD | 0.622(0.554 – 0.689) | 21.0 | 91.9% | 30.1% | 0.221 |
| pNN50 | 0.606(0.537 – 0.674) | 2.8 | 91.1% | 31.6% | 0.227 |
